# Distribution of SARS-CoV-2 Genomes in Wastewaters and the Associated Potential Infection Risk for Plant Workers in Typical Urban and Peri-Urban Communities of the Buffalo City Region, South Africa

**DOI:** 10.3390/v16060871

**Published:** 2024-05-29

**Authors:** Balisa Ngqwala, Luyanda Msolo, Kingsley Ehi Ebomah, Nolonwabo Nontongana, Anthony Ifeanyi Okoh

**Affiliations:** 1 SAMRC Microbial Water Quality Monitoring Centre, University of Fort Hare, Alice 5700, South Africa; lmsolo@ufh.ac.za (L.M.); kebomah@ufh.ac.za (K.E.E.); nnontongana@ufh.ac.za (N.N.); aokoh@ufh.ac.za (A.I.O.); 2 Applied and Environmental Microbiology Research Group (AEMREG), Department of Biochemistry and Microbiology, University of Fort Hare, Alice 5700, South Africa

**Keywords:** coronaviruses, COVID-19, infection risk, plant workers, SARS-CoV-2, wastewater

## Abstract

The presence of severe acute respiratory syndrome coronavirus 2 (SARS-CoV-2) in wastewater has been reported in several studies and similar research can be used as a proxy for an early warning of potential Coronavirus disease 2019 (COVID-19) outbreaks. This study focused on profiling the incidence of SARS-CoV-2 genomes in wastewater samples obtained from facilities located in the Buffalo City Municipality. Raw samples were collected weekly using the grab technique for a period of 48 weeks. Ribonucleic acids were extracted from the samples, using the QIAGEN Powersoil Total RNA Extraction kit, and extracted RNA samples were further profiled for the presence of SARS-CoV-2 genomes using Quantitative Real-Time Polymerase Chain Reaction (qRT-PCR) technique. Furthermore, various environmental matrices were utilized to estimate the potential health risk to plant operators associated with exposure to SARS-CoV-2 viral particles using the quantitative microbiological risk assessment (QMRA) model. Our findings revealed the prevalence of SARS-CoV-2 genomes with concentrations that ranged from 0.22 $\times \;10^{3}$ to 17.60 $\times \;10^{3}$ genome copies per milliliter (GC/mL). Different exposure scenarios were employed for the QMRA model, and the findings indicate a probability of infection (P(i)) ranging from 0.93% to 37.81% across the study sites. Similarly, the P(i) was highly significant (*p* < 0.001) for the 20 mL volumetric intake as compared to other volumetric intake scenarios, and high P(i) was also observed in spring, autumn, and winter for all WWTPs. The P(i) was significantly different (*p* < 0.05) with respect to the different seasons and with respect to different volume scenarios.

## 1. Introduction

Coronaviruses are a member of the Nidovirales order and Coronaviridae family and they are enveloped positive-sense, single-stranded RNA viruses [1]. These viruses not only constitute a threat to public health but also pose a risk to political and economic dimensions; moreover, they are contagious to humans, mammals, and avian species [1]. There are currently seven known human coronavirus (HCoV) strains include the following: 229E, OC43, HKU1, NL63, SARS-CoV, MERS-CoV, and SARS-CoV-2 currently, the coronavirus family is divided into four subgroups, namely, alpha, beta, gamma, and delta coronaviruses [2].

Coronaviruses encompass spike-like surface projections, a large unique genome, a characteristic self-replication phenomenon, and high rates of mutation and recombination [2]. These viruses must cross the species barrier and find a new host to survive and multiply because of these physiognomies. The four structural proteins of the virus are the nucleocapsid (N), membrane (M), Envelope protein (E), and spike (S). According to Baez-Santo et al. [1], the ability of the coronavirus spike protein (S) to accurately attach to the receptors of cellular entry, which has been discovered for several of these viruses, such as angiotensin-converting enzyme 2 (ACE2), is the initial step in coronavirus infection. Viral tissue distribution and entrance receptor expression determine viral tropism and pathogenicity. The host receptor for the spike (S) glycoproteins on its surface is ACE2 receptors [1]. Human ACE2 (hACE2) is recognized by numerous Coronaviruses and enters the cells via endocytosis.

Since its inception, SARS-CoV-2 has diverged into numerous clades and lineages based on distinctive mutational features [3]. Several subvariants have been identified since the Omicron variant first emerged in late 2021 such as BA.1, BA.2 BA.4, and BA.5. Due to the virus’ proliferation, the World Health Organization (WHO) has identified these variants as Variants of Concern (VOCs) [4]. At the end of 2019, a novel coronavirus outbreak occurred in Wuhan China, and, in the first fifty days of the pandemic, the virus reportedly caused more than 1800 fatalities and recorded more than 70,000 infections [5]. Since its first discovery in December 2019 to date, the virus has spread throughout the world, accounting for over 671 million cases of infection with more than 6.84 million cases of fatalities [6]. In South Africa alone, more than 102,000 deaths with over 4.06 million episodes have been reported [7].

According to Cahill and Morris [8], the main form of COVID-19 transmission is respiratory droplets from an infected person. Current evidence demonstrates the existence of the virus in the feces and urine of human beings [6]. Additionally, the possibility of the disease spreading through water, soil, and other environmental compartments raises concerns due to the presence of the virus in human urine and feces. Moreover, several studies have discussed the fecal–oral route as one of the important modes of transmission of SARS-CoV-2 [9]. According to Andier et al. [10], the survival of viruses in nasal, salivary, respiratory, or fecal secretions give some insights into the mechanisms by which weather might contribute to seasonality. Virus survival and distribution studies suggest that environmental variables, such as humidity, temperature, sunlight, and UV radiation, might contribute to the seasonal transmission of coronaviruses by influencing the inactivation of the virus in air and on surfaces. Another study by Liu et al. [11] suggested that all coronaviruses including SARS-CoV-2 tend to survive longer in colder and dryer conditions and enhance the virus’ ability to spread. According to Harmoto et al. [12], wastewater-based epidemiology (WBE) is the most advisable model to be used as an early indication or detection framework for future pandemics. This model includes the detection of nucleic acids through Polymerase Chain Reaction (PCR)-based techniques.

These PCR techniques offer high accuracy and explicitness; however, they require intricate sample processing within the laboratory, an exceptional aptitude, and an extensive stretch of information handling and investigation [13]. There are numerous documented studies on disease surveillance in wastewater using PCR techniques that have been recorded in several countries, including Australia, the Netherlands, Italy, Spain, France, Japan, the United States of America, Ecuador, India, and Germany [14]. The virus in the water cycle was thoroughly investigated, and the effectiveness of different treatment approaches at various phases of wastewater treatment plants in eliminating the virus was evaluated.

To reduce the likelihood of a new epidemic, future research perspectives on the thorough performance assessment of wastewater treatment procedures are offered. The Quantitative Microbial Risk Assessment (QMRA) model has been used to evaluate bioaerosols, drinking water, reclaimed water, recreational water, irrigation water, and wastewater for health hazards [15]. Recent studies have suggested the use of QMRA to evaluate the risk of the adverse effects of microbial infections linked with exposure to wastewater based on prior research on relevant respiratory viruses such as SARS-CoV and MERS-CoV [15].

## 2. Materials and Methods

### 2.1. Study Site Description

**Figure 1:**
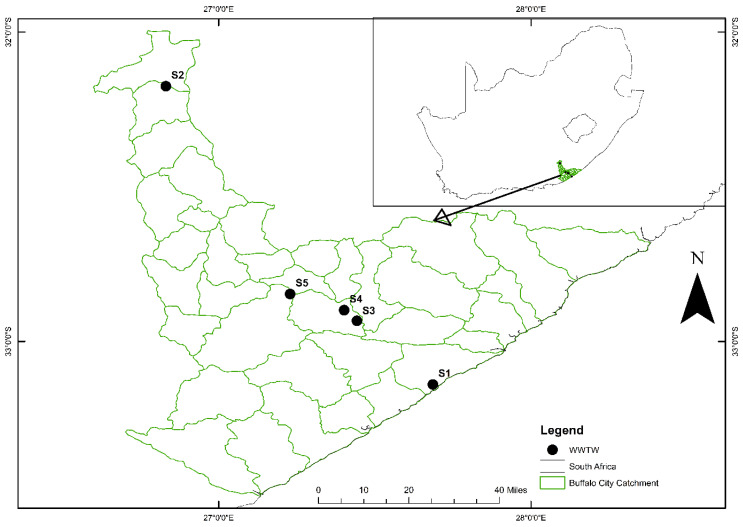
Geographical locations of selected wastewater treatment plants in Buffalo City Metropolitan Municipality. The WWTPs used in this study are located in S1, S2, S3, S4, and S5 in the Buffalo City Metropolitan Municipality in the Eastern Cape Province, South Africa. These plants discharge their final influents into the catchment areas listed in Table 1.

This study was conducted in Buffalo City Metropolitan Municipality (BCM) in the Eastern Cape Province, South Africa (Figure 1). According to the Eastern Cape Economic Consultative Council [16], a total population of 920,000 is accounted for by this Municipality, which is considered the most densely populated metropolitan municipality in the province, with an average of 408 people per square kilometer population density. This region has considerable challenges of poorly maintained infrastructures such as wastewater treatment plants and sanitation facilities [9]. During this study, the wastewater treatment plants under study were often overflowing, due to burst pipes and blockages; moreover, there were, consequently, frequently disseminated untreated effluents to the receiving watershed and the nearby environment, which exposed the public to an elevated health risk. Among the unpleasant practices notable in these sites, the plant operators performed their daily activities without wearing any personal protective equipment/gear (PPE), which exposed them to a risk of infection from the cocktail of potentially pathogenic agents in the wastewater, including SARS-CoV-2.

### 2.2. Sample Collection and Processing

Raw wastewater (influent) samples were collected on a weekly basis for a period of 48 weeks (1 June 2022 to 30 May 2023) using the grab sampling technique. The raw wastewater samples were collected in pre-sterilized 500 mL sampling bottles from the wastewater treatment facilities. Samplers wore standard personal protective equipment (PPE), such as disposable coveralls, appropriate boots, nose masks face shields, and latex hand gloves to ensure safe sample collection and to reduce the risk of exposure to SARS-CoV-2 viral particles. Alcohol-based hand sanitizers were constantly used prior to and after sample handling to avoid the transmission of pathogens. Collected influent samples were aseptically transported in ice in properly sealed cooler boxes to the SAMRC Microbial Water Quality Monitoring Laboratory at the University of Fort Hare for analysis not more than six hours after collection.

### 2.3. Viral RNA Extraction and Quantification

From the collected wastewater (WW) samples, viral RNA was extracted in a Biosafety Level 2 safety cabinet using the method described by Walsh et al. [18]. Briefly, aliquots of at least 100 mL of homogenized raw wastewater samples were centrifuged in conical tubes at 2500× *g* for 20 min. The supernatant was discarded, and about 5–7.5 mL of the collected pellet was utilized to extract the total RNA using the QIAGEN RNeasy PowerSoil Total RNA kit according to the manufacturer’s guidelines. The purity and concentration of the extracted RNA were determined using the Nanodrop^TM^ One Microvolume UV–Vis spectrophotometer (Thermo Fisher Scientific, Waltham, MA, USA).

### 2.4. Profiling of SARS-CoV-2 Genomes Using Quantitative Real-Time Polymerase Chain Reaction

iTaq Probe-Based qPCR assays (BIORAD Laboratories, Hercules, CA, USA) were utilized to detect and quantify the concentrations of SARS-CoV-2 genomes in the extracted RNA samples using the QuantStudio 5 qPCR system (Applied Biosystems, Waltham, MA, USA). The amplification and quantification of the virus genome was performed by amplifying the SARS-CoV-2_N1-P and SARS-CoV-2_N2-P (2019-nCoV, CDC, EUA) target genes of the Nucleocapsid protein of the virus.

The qPCR was performed in a 96-well 0.2 mL block reaction plate (Applied Biosystems^®^) by aliquoting 1 μL of the total RNA extracts to 9 μL of the RT-qPCR reaction mixture consisting of a 5 μL iTaq universal probes reaction mix, 0.25 μL of iScript RT advanced reverse transcriptase (BIORAD, USA), 0.5 μL of each SARS-CoV-2_N1-P and SARS-CoV-2_N2-P, and 3.25 μL of sterile Nuclease free water (BIORAD, USA), making up a final volume of 10 μL per reaction. The reaction plate was carefully sealed with adhesive covers. All qPCR procedures ran for 40 cycles in the QuantStudio 5 qPCR system (Applied Biosystems) followed by analysis using the Design and Analysis 2.4 RT PCR Software (Applied Biosystems^®^). The description of oligonucleotide sequence and RT-PCR cycling conditions can be seen in a recent study by Qongwe et al. [19]. An analysis to obtain the quantitative data was performed on each well, where positive samples were determined by the average Cycle Threshold (CT) values ≤ 35 cycles, while the limit of detection threshold was set at 0.02 per reaction.

### 2.5. Quantitative Microbial Risk Assessment of SARS-CoV-2 Genomes among Wastewater Treatment Plant Operators

Various environmental matrices have been utilized to estimate hazards to human health from exposure to pathogens using the quantitative microbiological risk assessment (QMRA) technique. It has characteristic features that need to be considered including the workers’ activities as well as the exposure and transmission pathways. QMRA was carried out to estimate the potential risks posed by the SARS-CoV-2 genome to wastewater treatment plant workers using the following harmonized framework as described by Carducci et al. [15]:

I. **Hazard Identification:** During previous coronavirus outbreaks, the generation of wastewater aerosols and droplets was verified as a crucial mechanism of fecal respiration transmission, and this was also suspected in the ongoing COVID-19 outbreak brought on by SARS-CoV-2 [20]. SARS-CoV-2 incident was identified through an exact number of SAR-CoV-2 viral copies from the positive samples.
II. **Exposure Assessment:** This describes the characteristic pathways that allow SARS-CoV-2 to spread to people and cause infection. Based on assumptions, the wastewater treatment plant operators were exposed to an aerosolized form of the virus through wind when performing their daily duties such as sampling, manual cleaning, and course screening [21]. These workers were assumed to be present at the wastewater treatment plant site for a period of 6 h a day with different volumetric intakes that were exposed to represent the low-case, moderate-case, and worst-case scenarios. For exposure assessment, the SARS-CoV-2 loads in raw wastewater were used to evaluate the level of risk of infection due to the presence of SARS-CoV-2 in the influents. However, the average viral copies/mL were converted into doses and reference was made to inhalation rates in a study by Dada and Gyawali [21], using 3 different scenarios with a volumetric intake of 2, 10, and 20 mL.

d = I × VC(1)

where VC = seasonal average concentration of viral load in raw wastewater influents (genomic copies/mL), I = volumetric intake, and d = dose

In order to estimate d, the volumetric intake was multiplied by VC. Then, the genome copies per mL were converted to a plaque-forming unit (PFU). According to literature 1000 genomic copies per mL = 1 PFU [21,22].

III. **Dose Response:** Previously conducted QMRAs show that dose-response information for other pathogens is often lacking; hence, that of SARS-CoV-2 does not exist. As such, SARS-CoV-1 was used as a substitute to determine the probability of SARS-CoV-2 concentration to cause an infection with a k constant (4.1 × 10^2^) [23,24]. Dose-response determines the probability of the concentration of the virus to cause an infection using a mathematical model with an exponential model equation that is described as follows:

(2) $$P(i)=1-e^{\left(-\frac{d}{k}\right)}$$

d = I × VC

where P(i)—probability of infection after a single dose

d—dose, as number of organisms ingested (PFU)

k—exponential constant (4.1 × $10^{2}$)

I—volumetric intake (2 mL, 10 mL, 20 mL)

VC—concentration of viral load in raw wastewater influents (genomic copies/mL)

The probability of infection was determined under three different scenarios with wastewater treatment plant operators being present at the wastewater treatment site for a period of 6 h, with a volumetric intake of 2, 10, and 20 mL. The factors that influence the risk estimates were mostly related to the dose-response pathogen. After d was determined and converted to PFU, variables were then substituted into the equation to obtain the probability of infection.

### 2.6. Statistical Analysis

Statistical analysis was determined by using Bayesian One-way ANOVA and the general linear model (Univariate) on IBM SPSS Statistics Data Editor to compare the level of significance.

## 3. Results

### 3.1. Viral RNA Extraction and Quantification

**Figure 2:**
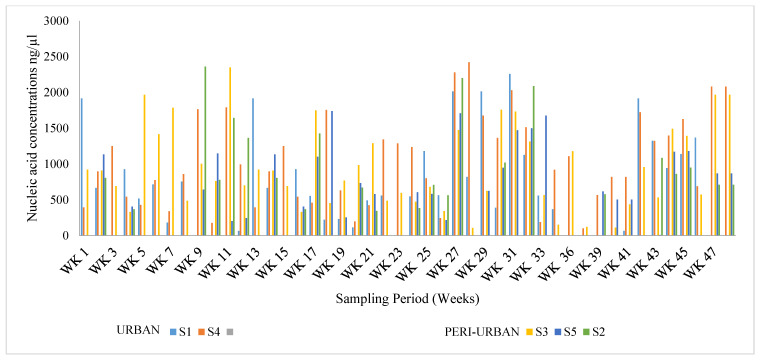
Graphical representation of the total nucleic acid concentration extracted from Buffalo City Region selected wastewater treatment plants throughout the weekly sampling regime. Reasonably high nucleic acid concentrations were obtained throughout the sampling regime.

Total viral ribonucleic acids were extracted from a total of 100 mL processed wastewater samples over a period of 48 weeks. The extracted nucleic acid concentrations in Figure 2 revealed high concentrations between week 9 and week 13 more than 2.36 × ${\;10}^{3}$ ng/µL, a significant decline in comparison with week 15 and week 17. Additionally, an increase in the total RNA concentration was subsequently observed between week 27 and week 33, with 2.48 $\times \;10^{3}\;ng/µL$ and 2.25 × $10^{3}\;ng/µL,$ respectively. This fluctuation is observed in low-income communities or peri-urban areas such as S3, S2, and S5, in which inadequately treated wastewater was occasionally released directly into the environment due to dysfunctional treatment plants in these sites and could potentially contaminate the groundwater. The majority of households in these areas utilize groundwater for various needs such as drinking, cooking, laundry, and other recreational purposes, resulting in potential SARS-CoV-2 viral transmission through infected and untreated groundwater as observed in S3 and S2 on the stipulated weeks, with approximately 2.34 × $10^{3}\;ng/µL$ and 2.36 $\times \;10^{3}\;ng/µL,$ respectively. Although these concentrations represent the total RNA in a sample, a similar peak in the COVID-19 clinical cases from these sites was observed during the same period with a recorded total number of 2,511,178 and 2,889,298 infections, respectively [23].

According to Arslan et al. [24], SARS-CoV-2 can survive for approximately four days in diarrheal stool samples with alkaline pH, and more than seven days in respiratory samples at room temperature. In a recent study, Qongwe et al. [19] reported that the fecal–oral transmission of SARS-CoV-2 is established in low-income countries that have poor sanitation, which makes sewage and surface waters important sources of dissemination of SARS-CoV-2. Likewise, the high concentrations of total RNA observed in Figure 2 from the various sites in this present study further accentuate the concept highlighted by Haramoto and colleagues.

### 3.2. Profiling of SARS-CoV-2 Genomes Using Quantitative Real-Time Polymerase Chain Reaction (qRT-PCR)

The amplification and quantification of the virus in the processed raw wastewater samples was accomplished by amplifying the SARS-CoV-2_N1-P and SARS-CoV-2_N2-P (2019-nCoV, CDC, EUA) target genes of the nucleocapsid protein in coronaviruses.

On average, the resultant standard curve slope for N1 and N2 generated from the qPCR reaction was −3.575 and −3.432, respectively, with the amplification efficiency of 90.427% and 95.608%, which fell within the desired recommended amplification efficiency from 90% to 105%, indicating the high-profile sensitivity of the reaction. Theoretically, the acceptable standard curve slope should fall within the range of −3.3 to −3.6; therefore, these qPCR outcomes complied with the standard desirable slope range. Moreover, the standard error of the experiment was as low as 0.078 and 0.302 for the N1 and N2 targets, respectively, which is in line with the standard requirement that the standard error of the slope is as close to zero as possible. The data were then exported to Microsoft Excel to further quantify the exact number of SAR-CoV-2 viral copies/mL from the positive samples. The data acquired from qPCR amplification was used to calculate the exact genome copies per mL (GC/mL) using the following formula:

Average quantity × dilution factor (10) = Viral copy number/µL

**Figure 3:**
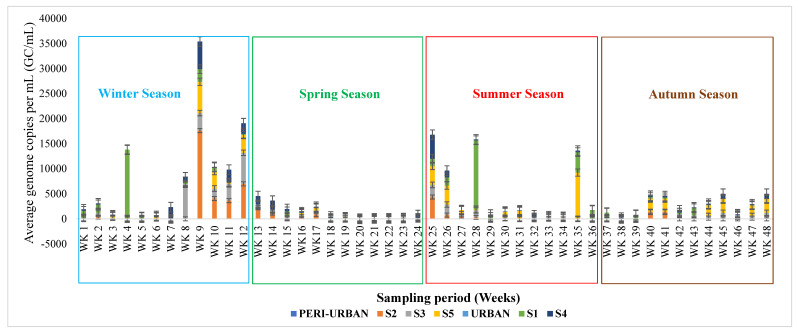
Graphical representation of the SARS-CoV-2 N1/N2 average genome copies/mL detected across the 5 study sites throughout the surveillance period. This figure also represents the seasonal distribution of SARS-CoV-2 from selected Buffalo City Metropolitan Municipality wastewater treatment plants during winter, spring, summer, and autumn seasons.

Genome copies per mL (GC/mL) = (copy number/µL × 1000)/volume of the extracted sample. Figure 3 below represents the obtained SARS-CoV-2 N1/N2 average copy numbers/mL across the five study sites throughout the surveillance period.

According to Wu et al. [25], the prolonged fecal excretion of SARS-CoV-2 RNA was reported for up to seven weeks after the onset of initial symptoms. Another clinical study detected SARS-CoV-2 in approximately 82% of fecal samples of infected individuals [6]. In the present study, the N1 and N2 regions, which are contained within the nucleocapsid gene, were the targets for SARS-CoV-2 RNA detection in wastewater. The viral nucleocapsid gene is the most important transcript of SARS-CoV-2 RNA and is, therefore, commonly utilized for the environmental monitoring of the virus [26].

Overall, the findings of this study indicate the presence of SARS-CoV-2 RNA from the influent wastewater samples collected from S3, S4, S5, S2, and S1 wastewater treatment facilities with virus concentrations of 3.49 ${\times \;10}^{3},$ 2.32 $\times \;10^{3}$, 6.25 × $10^{3}$, 6.$96\times \;10^{3}$ and 1.45 $\times \;10^{3}$ genome copies per milliliter (GC/mL) per week, respectively. As outlined in Figure 3, the highest viral loads were obtained between week 8 and week 12 (from the end of July–August 2021) from sites S2, S3, and S4. This drastic rise may be attributed to the emergence of the delta variant, which was more transmissible during this period because of gene mutations that are found in the spike proteins and drove the third wave of infections in South Africa [27]. This variant had at least 21 characteristic mutations [26]. Similarly, using clinical data from confirmed COVID-19 cases in the regions with 1,995,556 and 2,680,225 confirmed cases, WHO [4] revealed a significant correlation with virus concentrations, which corroborates the findings of this study.

The most notable gene mutations that are suspected to enable the delta variant to be the most transmissible variant are found in the spike proteins. The spike gene mutations in the B.1.617.2 variant include T19R, L452R, T478K, D614G, P681R, and d960N, with deletions at positions 157 and 158 [28]. The D614G and L452R, increase the stability of the RBD–ACE2 complex and increase infectivity while P681R improves spikes protein cleavage and increases transmissibility [27]. All these mutations provided a higher infective capability of the virus than the previously reported variants [4].

The National Centre for Disease Control (NCDC) [29] reported a seven-day average increase of over 69% in new COVID-19 cases with a 35% increase in hospitalizations [30]. This report corroborates the findings of this study as a similar increase in viral genome copies was observed around the same time. Additionally, significant factors that influence the number of viral genome copies of SARS-CoV-2 in wastewater include the number of SARS-CoV-2-infected individuals in a catchment and the amount of viral RNA that COVID-19 patients shed; hence, we observed the fluctuation outlined in Figure 2 and Figure 3.

The high viral loads obtained in the peri-urban facilities such as in sites S2 and S3 may be due to the high population size of the communities being served by these facilities. Another contributing factor could be the poor or complete lack of maintenance of infrastructure within these wastewater collection and treatment facilities, which, subsequently, results in the circulation and dissemination of the virus in the surrounding environment.

Recently, the discharge of sewage into receiving watersheds has been reported to be the potential route of transmission for SARS-CoV-2 [6]. However, Castiglioni et al. [31] documented that SARS-CoV-2 detected in raw sewage was not infectious. Another study conducted in France reported a directly proportional relationship between the increase in the number of COVID-19 cases and RNA viral load from untreated sewage in the region [26]. Similar studies conducted in the United States of America, and Australia reported the prevalence of viral RNA fragments in untreated wastewater samples with a maximum concentration of over ${\;10}^{9}$ copies/mL [25,26,32]. The above studies corroborate the findings of the present study as high concentrations of RNA fragments were obtained in sites S2 and S1, with over 17 ×${\;10}^{3}$ and 13 ×${\;10}^{3}$ viral genome copies/mL, respectively.

The findings of this study support the need for regular wastewater monitoring as an additional strategy to current tracking methods utilized to mitigate COVID-19 spread. Nonetheless, there are a few noticeable limitations in the present study. For instance, it is mostly impossible to estimate the disease burden of the population based on wastewater testing for SARS-CoV-2 because of numerous sources of variability, including the concentration of SARS-CoV-2 excretion by infected individuals and variations in the rate of degradation of viral RNA in wastewater [33]. However, the routine sampling strategy adopted in this study allowed for seasonal comparisons of viral RNA concentrations to be achieved in each wastewater treatment plant, ensuring reliable data updates on the changes detectable from the weekly sampling regime. Figure 3 represents the seasonal distribution of SARS-CoV-2 RNA fragments across the selected wastewater treatment facilities within the Buffalo City Metropolitan Municipality.

A key component in the transmission of the disease has been the SARS-CoV-2 variants of concern (VOCs). As observed in Figure 3, during winter (week 7 to week 12), and from the end of spring to mid-summer (week 24 to week 29), a peak in the viral loads averaged at 5.86 × $10^{3}$ genome copies/mL was obtained. This is the same period when the Omicron variant was dominant and circulated excessively in South Africa, driving the fourth wave of infections [34]. This variant was highly transmissible and, hence, resulted in an increased number of infections even though it had lower hospital admissions and less severity because of the introduction of vaccines among civilians.

According to Han et al. [35], the presence of SARS-CoV-2 in wastewater poses an additional concern in communities served by combined sewer systems, as urban flooding, a common hazard during rainy seasons, can lead to sewage overflow, posing a new risk for the spread of SARS-CoV-2 in the impacted areas. This is evident in Figure 3 between epidemiological week 27 and week 29. This may also elucidate the drastic decline in the viral load obtained since there were no samples to profile the highlighted weeks due to damaged plant stations by the storm surge.

### 3.3. Quantitative Microbial Risk Assessment of the SARS-CoV-2 Genomes

**Figure 4:**
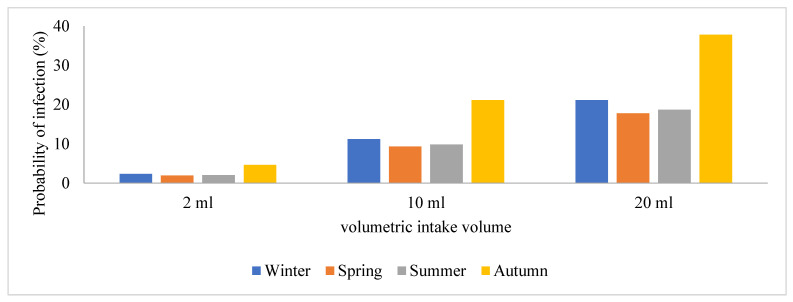
Graphical representation of the probability of infection among the plant operators in the wastewater treatment facility located in S4.

**Figure 5:**
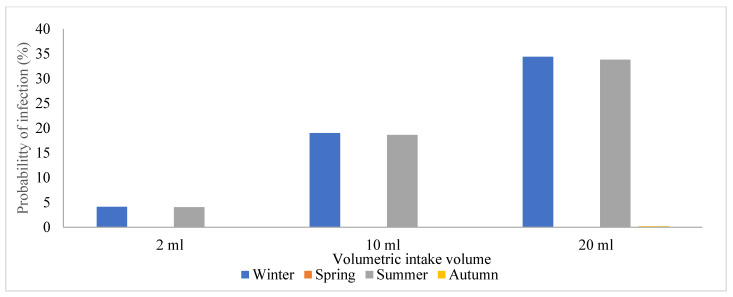
Graphical representation of the probability of infection among the plant operators in the wastewater treatment facility located in S1.

**Figure 6:**
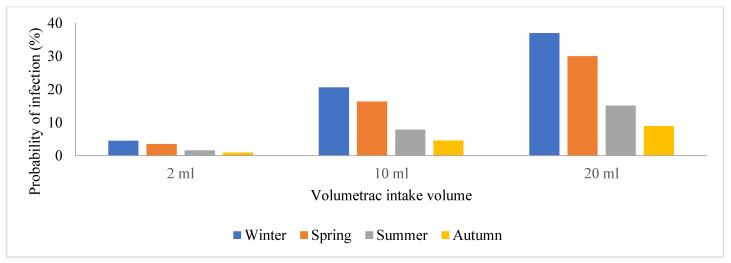
Graphical representation of the probability of infection among the plant operators in the wastewater treatment facility located in S2.

**Figure 7:**
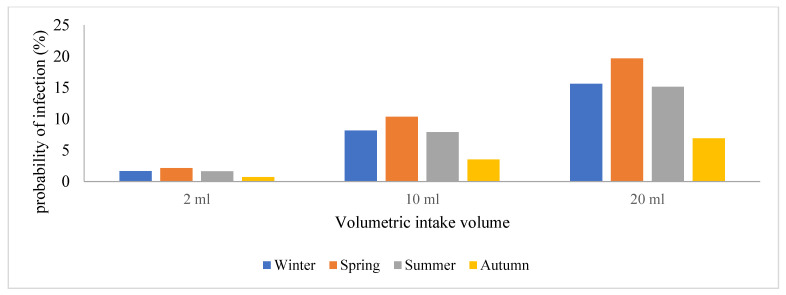
Graphical representation of the probability of infection among the plant operators in the wastewater treatment facility located in S5.

Due to the prevalence of SARS-CoV-2 in these areas, wastewater treatment plant operators are exposed to a significant risk of contracting the viral particles while performing their regular duties or routine maintenance. Hence, this study sought to estimate and assess the potential risks posed by SARS-CoV-2 to human health by conducting a quantitative microbiological risk assessment (QMRA). In this study, an exponential model for SARS-CoV-1 and the dose-response model with a k constant (4.1 × $10^{2}$) were used, and we generated a graph of volumetric intake against P(i) across the four seasons in Figure 4, Figure 5, Figure 6 and Figure 7. This model took a conservative approach to be protective of worker’s health by estimating the levels of potential risks posed by SARS-CoV-2 to their health, assuming different exposure scenarios [21]. Since SARS-CoV-2 incidence was identified through genome copies, it was assumed that aerosolized SARS-CoV-2 viral particles were introduced to wastewater treatment plant operators when performing their daily activities such as wastewater sampling and course screening.

## 4. Discussion

**Figure 8:**
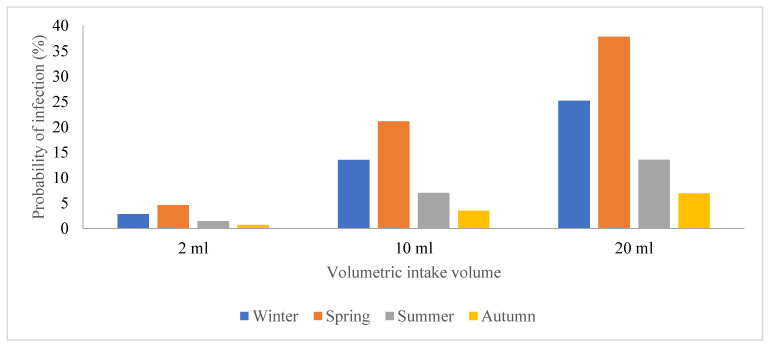
Graphical representation of the probability of infection among the plant operators in the wastewater treatment facility located in S3.

**Figure 9:**
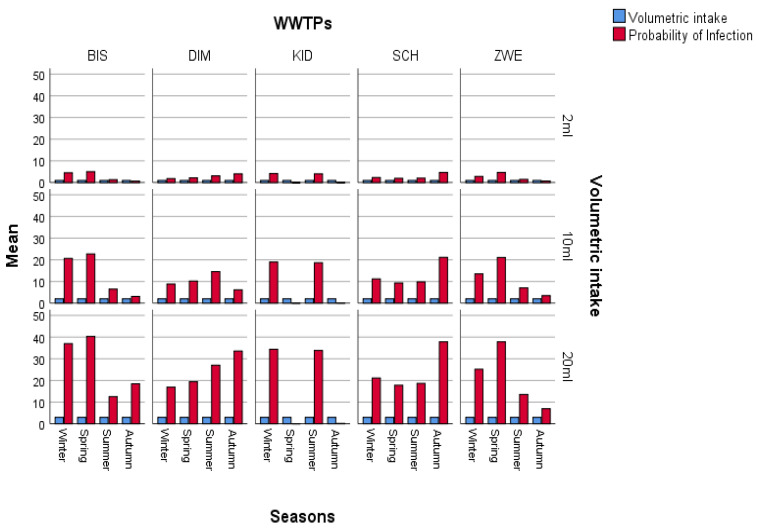
A bar plot showing the frequency of P(i) among different WWTPs, highlighting the effect of seasons as compared with the different volumetric intake scenarios.

Previous QMRA studies have shown that a dose-response model for SARS-CoV-2 does not exist; hence, a dose-response model for SARS-CoV-1 was adopted and used as a substitute because of their similarities in structure and their ability to cause an infection, and this exponential model was used to determine the probability of SARS-CoV-2 particles to cause an infection on the wastewater treatment plant operators [15]. This is illustrated in Figure 5, Figure 6, Figure 7, Figure 8 and Figure 9, in which the probability of infection depends on the viral loads in that particular site and is expressed by the higher values of the probability of infection. Bayesian One-way ANOVA and general linear model (Univariate) were used on the IBM SPSS Statistics Data Editor to compare the level of significance with respect to the different seasons and with respect to different volumetric scenarios. The P(i) was highly significant (*p* < 0.001) for 20 mL as compared to other volume scenarios (Figure 9) and high P(i) was also observed in spring, autumn, and winter for all WWTPs. However, according to the Duncan plot, seasons were 0.01% significant on P(i). Hence, the P(i) was significantly different (*p* < 0.05) with respect to the different seasons as well as the different volumetric intake scenarios. Supplementary Table S1 summarizes the probability of infection, significance, and subset across all WWTPs from the different models used for statistical analysis. However, given varied assumptions and restrictions, the projected risk levels derived may have significant uncertainty, as determining exposure assessment for occupational risks in the sites is particularly a challenge due to the wide range of activities as well as seasonal changes. The fecal–oral route of transmission in low-income areas with insufficient sanitation infrastructure poses a major health hazard since untreated waste with potentially infective SARS-CoV-2 viral particles subsequently sets residents within the catchment area at a high risk of contracting the virus. SARS-CoV-2 infectivity results in COVID-19 dissemination in these environments, which remains a major global health concern to date.

This study evaluated the presence and distribution of SARS-CoV-2 genomes in wastewater samples from some urban and peri-urban wastewater treatment facilities in the Buffalo City Municipality, Eastern Cape Province, South Africa. SARS-CoV-2 genomes were quantified using Real-Time PCR. The Quantitative Real-Time Polymerase Chain Reaction analysis remains the gold standard for the accurate and timeous detection of SARS-CoV-2 genomes in biological and environmental specimens, because of its sensitivity and efficacy. The presence of SARS-CoV-2 genomes in wastewater samples signals a fecal–oral route of transmission and subsequent dissemination of SARS-CoV-2 viral particles into the wastewater treatment facilities. Data obtained in the present study confirm the presence of SARS-CoV-2 genomes in the wastewater samples. Moreover, the WWTPs disseminate SARS-CoV-2 genomes to the receiving watersheds, as most of these facilities were very often overflowing, thereby releasing untreated sewage to the environment, thus constituting a public health risk.

## 5. Conclusions

The high SARS-CoV-2 genome load obtained in the peri-urban wastewater treatment plants (WWTPs) may be due to the highly dense population served by these facilities, and to a greater extent, the lack of quality sanitary infrastructures in the regions. Furthermore, the poorly maintained WWTPs in the Eastern Cape Province continue to be a bothersome public and environmental health burden. WWTP workers and operators are at greater risk during their daily activities, and, consequently, this study lays emphasis on the potential health risks that plant operators encounter when performing their daily duties. Nonetheless, the level of risk generated is highlighted by the level of significance with respect to different volumetric scenarios, as the probability of infection is directly proportional to the volumetric intake. Frequent monitoring of the aquatic matrices and wastewater-based epidemiology (WBE) studies both provide early warning signals for the proliferation of the SARS-CoV-2 and other microbial pathogens, and more surveillance studies can aid in mitigating the public health threat brought upon by these pathogens.

## Figures and Tables

**Table 1:** Catchment areas, population density, and technology use of the study sites [17].

Site	Capacity of the Plant (Megaliters/Day)	Technology Use of Wastewater Treatment Plants	Area (Coverage)	Population Density	Population	Catchment Area
**S1**	0.4	Stabilization pond	2.130 km^2^	230.0/km^2^	69,900	Mcantsi river
**S2**	2	Stabilization pond	8.082 km^2^	1400/km^2^	11,192	Yellowwoods river
**S3**	7.5	Bio-filters	4.646 km^2^	3900/km^2^	18,189	Buffalo river
**S4**	7	Activated sludge model	65.52 km^2^	520/km^2^	34,019	Buffalo river
**S5**	8	Activated sludge model	17.289 km^2^	1300/km^2^	21,783	Mdizeni stream

## Data Availability

Data-sharing inquiries should be directed to the authors.

## Supplementary Materials

The following supporting information can be downloaded at: https://www.mdpi.com/article/10.3390/v16060871/s1. Table S1: the probability of infection, significance, and subset across all WWTPs.
