# Supplementary material for: Distribution of SARS-CoV-2 Genomes in Wastewaters and the Associated Potential Infection Risk for Plant Workers in Typical Urban and Peri-Urban Communities of the Buffalo City Region, South Africa
Source: Viruses. 2024 May 29;16(6):871. doi: 10.3390/v16060871 (PMC11209190; doi:10.3390/v16060871)
Supplement: Supplementary file 1 [file viruses-16-00871-s001.zip › viruses-3000761-supplementary.pdf]

**Table S1.** Probability of infection, significance, and subset across all WWTPs.

| **Probability of Infection** | | | | | |
| --- | --- | --- | --- | --- | --- |
| | **Volumetric Intake** | **N** | **Subset** | | |
| | | | 1 | 2 | 3 |
| Student–Newman–Keuls[a,b] | 2 mL | 20 | 2.5716 | | |
| | 10 mL | 20 | | 11.363 | |
| | 20 mL | 20 | | | 22.6305 |
| | Sig. | | 1 | 1 | 1 |
| Tukey HSDa,b | 2 mL | 20 | 2.5716 | | |
| | 10 mL | 20 | | 11.363 | |
| | 20 mL | 20 | | | 22.6305 |
| | Sig. | | 1 | 1 | 1 |
| Tukey Ba,b | 2 mL | 20 | 2.5716 | | |
| | 10 mL | 20 | | 11.363 | |
| | 20 mL | 20 | | | 22.6305 |
| Duncana,b | 2 mL | 20 | 2.5716 | | |
| | 10 mL | 20 | | 11.363 | |
| | 20 mL | 20 | | | 22.6305 |
| | Sig. | | 1 | 1 | 1 |
| Scheffea,b | 2 mL | 20 | 2.5716 | | |
| | 10 mL | 20 | | 11.363 | |
| | 20 mL | 20 | | | 22.6305 |
| | Sig. | | 1 | 1 | 1 |
| Gabriela,b | 2 mL | 20 | 2.5716 | | |
| | 10 mL | 20 | | 11.363 | |
| | 20 mL | 20 | | | 22.6305 |
| | Sig. | | 1 | 1 | 1 |
| Ryan–Einot–Gabriel–Welsch Fb | 2 mL | 20 | 2.5716 | | |
| | 10 mL | 20 | | 11.363 | |
| | 20 mL | 20 | | | 22.6305 |
| | Sig. | | 1 | 1 | 1 |
| Ryan–Einot–Gabriel–Welsch Rangeb | 2 mL | 20 | 2.5716 | | |
| | 10 mL | 20 | | 11.363 | |
| | 20 mL | 20 | | | 22.6305 |
| | Sig. | | 1 | 1 | 1 |
| Hochberga,b | 2 mL | 20 | 2.5716 | | |
| | 10 mL | 20 | | 11.363 | |
| | 20 mL | 20 | | | 22.6305 |
| | Sig. | | 1 | 1 | 1 |
| Waller–Duncana,c | 2 mL | 20 | 2.5716 | | |
| | 10 mL | 20 | | 11.363 | |
| | 20 mL | 20 | | | 22.6305 |
| Means for groups in homogeneous subsets are displayed. | | | | | |
| Based on observed means. | | | | | |
| The error term is Mean Square (Error) = 69.355. | | | | | |
| a. Uses Harmonic Mean Sample Size = 20.000. | | | | | |
| b. Alpha = 0.05. | | | | | |
| c. Type I/Type II Error Seriousness Ratio = 100. | | | | | |
